# A Longitudinal Examination of Heart-Rate and Heart Rate Variability as Risk Markers for Child Posttraumatic Stress Symptoms in an Acute Injury Sample

**DOI:** 10.1007/s10802-019-00553-2

**Published:** 2019-05-10

**Authors:** Katharina Haag, Rachel Hiller, Peter Peyk, Tanja Michael, Richard Meiser-Stedman, Pasco Fearon, Anke Ehlers, Sarah L. Halligan

**Affiliations:** 1 Department of Psychology, University of Bath, Bath, UK; 2 Department of Consultation-Liaison-Psychiatry and Psychosomatic Medicine, University Hospital Zurich, Zürich, Switzerland; 3 Department for Clinical Psychology and Psychotherapy, University of Saarland, Saarbrücken, Germany; 4 Faculty of Medicine and Health Sciences, University of East Anglia, Norwich, UK; 5 Research Department of Clinical, Educational and Health Psychology, University College London, London, UK; 6 Department of Psychology, University of Oxford, Oxford, UK; 7 Department of Psychiatry and Mental Health, University of Cape Town, Cape Town, South Africa

**Keywords:** Longitudinal, Child, Adolescent, Posttraumatic stress disorder, Heart-rate, Heart-rate variability

## Abstract

Heart rate (HR) alterations in the immediate aftermath of trauma-exposure have been proposed to be potentially useful markers for child and adolescent posttraumatic stress disorder (PTSD). However, it is not yet clear if this holds true for measures taken more distal to the trauma, and no studies have investigated the predictive validity of more sensitive HR variability (HRV) indices. We recruited 76 parent-child pairs (child age 6 to 13 years) after the child experienced a traumatic event leading to presentation at a hospital emergency department. At 1-month post trauma (T1), HR recordings were obtained at rest, and while children verbally recounted their traumatic experience, both alone and together with a parent. Child post-traumatic stress symptoms (PTSS) were assessed concurrently (T1), and at 3 (T2) and 6-month (T3) follow-ups. We found that for T1, elevated mean HR during trauma narratives, but not at baseline, was positively associated with PTSS, with some evidence that HRV-indices were negatively cross-sectionally associated with PTSS. Furthermore, T1 HR indices predicted PTSS at T2 and partially at T3, although these effects did not hold when T1 PTSS were added to the model. Findings suggest that, consistent with the adult literature, HR indices in children may be a concurrent marker of higher PTSS and may be predictive of longer term distress. The findings encourage further investigations that track child HR and HRV in relation to PTSS over time after trauma, in order to examine how biological profiles evolve in those with persistent symptoms.

**Electronic supplementary material:**

The online version of this article (10.1007/s10802-019-00553-2) contains supplementary material, which is available to authorized users.

## Introduction

Meta-analytic reviews have found that between 10 and 20% of children and young people (CYP) will develop PTSD after exposure to traumatic events (Alisic et al. 2014; Hiller et al. 2016). The first 6 months post-trauma may be particularly important, as a period during which initial distress may remit spontaneously or become entrenched (Hiller et al. 2016). Since PTSD is known to have serious and persistent negative consequences for future adjustment (Salmon and Bryant 2002; Bolton et al. 2000), it is important to establish at the earliest possible stage which young people are at risk of persistent symptoms following trauma exposure, and to understand the processes via which risk is conferred. One way of achieving this is to identify biomarkers that are associated with the onset and progression of the disorder (Beauchaine and Thayer 2015).

Heart rate (HR) measures have been proposed to be potentially useful PTSD biomarkers, as they are relatively easy to obtain (Mayeux 2004; Olsson et al. 2008). Mechanistically, it has been hypothesized that HR elevations commonly found after trauma exposure may reflect the adrenergic response at the time of the event, as well as ongoing physiological stress reactions (Bryant 2006). As such, they are believed to influence key PTSD-relevant processes, such as initial fear conditioning and trauma memory consolidation, and to be related to ongoing symptoms including heightened distress in response to trauma reminders and generalised hyperarousal.

In adults, there is consistent evidence that elevated basal HR and heightened HR reactivity, particularly to trauma cues, are concurrently associated with PTSD, suggesting heightened sympathetic activation (Buckley and Kaloupek 2001). In addition, elevated basal HR assessed shortly after exposure to a range of traumatic events relatively consistently predicts higher PTSS up to 24 months later (meta-analytic weighted *r* = 0.13) (Morris et al. 2016). Studies of adult PTSD have also examined HR variability (HRV), which assesses changes in inter-beat intervals over time, and has been proposed to provide a more sensitive index of the autonomic stress response than mean HR (e.g. Appelhans and Luecken 2006).

Generally, higher HRV is believed to reflect a wider repertoire of responses to stress, while lower variability may reflect a diminished capacity to cope (Kim et al. 2018). Consequently, reduced HRV has been associated with decreased self-regulation and motivation for social interactions (Kemp and Quintana 2013), with psychological disorders including anxiety and depression (Bassett 2016; Chalmers et al. 2014), and with long term consequences such as higher cardiovascular disease and increased mortality (Thayer et al. 2010). One measure which is considered to be a reliable and interpretable index of HRV is Fourier-derived high frequency band power (HFBP, 0.15–0.4 Hz) (Billman 2013). HFBP is believed to reflect vagally-modulated functioning of the parasympathetic nervous system (PSN), with a higher HFBP indicating higher HRV, and thus a more adaptive stress response (De Bellis and Putnam 1994; Shaffer and Ginsberg 2017). A second, but less frequently studied measure of HRV, is low-frequency band power (LFBP, 0.04–0.15 Hz). While its interpretability is still debated due to a mix of sympathetic and parasympathetic influences, as basal LFBP is often reduced after trauma exposure, subsequent increases have been proposed to reflect the restoration of the sympatho-vagal balance (Nagpal et al. 2013; Reyes del Paso et al. 2013; Shaffer and Ginsberg 2017). A recent meta-analysis showed that in adults with PTSD relative to controls, there is consistent evidence for decreased basal HFBP, and for small, but significant reductions in basal LFBP (Nagpal et al. 2013), indicating HRV as a promising risk marker for PTSD.

In contrast to robust evidence of persistent cardiovascular alterations in association with PTSD in the adult field, studies of CYP have yielded less consistent findings. To date, the evidence has focused primarily on investigating mean HR. A number of cross-sectional studies have compared basal HR between PTSD and non-PTSD samples of CYP, recruited months to years after the original incident (for a review, see Kirsch et al. 2011). However, unlike the relatively consistent pattern of elevated basal autonomic activation found in adult PTSD, there is little evidence of equivalent changes in CYP. Across studies examining a range of age groups and trauma types, all but one (Scheeringa et al. 2004) found no difference in resting HR between CYP with PTSD and trauma-exposed and non-exposed controls (Gray et al. 2018; Jones-Alexander et al. 2005; Kirsch et al. 2015). Although much of this research has been limited by modest sample sizes, one study of 247 very young children aged 3–6 years also found no basal HR elevations in association with PTSD (Gray et al. 2018). Given the challenges of measuring PTSD in younger children, replication of the latter result in older children is desirable.

Other studies have focused on HR reactivity, rather than basal HR, as a PTSD marker in CYP. Using a range of non-trauma related provocations, no differences in HR reactivity were found between trauma-exposed young people with and without PTSD across diverse tasks (Grasso and Simons 2012; Gray et al. 2018; Jones-Alexander et al. 2005; Saltzman et al. 2005; Scheeringa et al. 2004; MacMillan et al. 2009). However, research focused on trauma-specific provocations has yielded more mixed findings. Thus, the aforementioned study of children aged 3–6 years found a significantly stronger decline in respiratory sinus arrhythmia (RSA) during trauma recall in those who suffered from PTSD, as compared to trauma-exposed and non-exposed controls (Gray et al. 2018). RSA is proposed to comprise a measure of parasympathetic tone that relates to emotional regulation (Beauchaine 2015). A second study of 124 pre-school children found higher HR reactivity to a trauma stimulus in trauma-exposed as compared to non-exposed children, but no differences between those with and without PTSD (Scheeringa et al. 2004). In contrast, two small studies that used trauma scripts as a provocation found that HR reactivity in CYP aged 7–18 with PTSD did not differ from either trauma exposed or non-exposed controls (Jones-Alexander et al. 2005; Kirsch et al. 2015).

In addition to cross-sectional evidence, key longitudinal studies have looked at the predictive value of HR in relation to PTSD in CYP (Kassam-Adams et al. 2005). There is relatively consistent evidence that elevated basal HR obtained within 24 h of emergency department (ED) admission following trauma-exposure is a positive predictor of short and long-term PTSS in CYP, with a meta-analysis of six studies yielding a small but reliable effect (*weighted r* = 0.18) (Alisic et al. 2011). However, there has been little longitudinal study of HR assessments taken more than 24 h after ED admission. One study found that HR indices assessed at hospital discharge after accidental trauma did not predict 6 month PTSS in CYP (Nugent et al. 2006). Learning more about the predictive value of HR measured more distal to the trauma is potentially important, as measures obtained early after trauma exposure could be confounded by factors such as pain and injury severity, and may not be available for all CYP.

In sum, while longitudinal studies indicate that elevated HR immediately following injury can predict later PTSS, evidence of more persistent changes is limited. In contrast to the adult literature, generally elevated autonomic activity has not been found in CYP with PTSD, and evidence as to whether or not there is greater reactivity to trauma stimuli is mixed. In addition, HRV may provide a more sensitive marker of the autonomic stress response but has been little investigated in CYP. In order to address gaps in the extant literature, we examined whether HR indices obtained 1 month after child trauma exposure were associated with PTSS observed concurrently, and whether they were predictive of persistent PTSS 3 and 6 months later, in a sample of 76 children aged 6–13 years. We examined both mean HR and HRV (HFBP, LFBP) indices, obtained at rest and while the children provided narratives of the traumatic experience a) alone and b) together with their parents. We hypothesized that child mean HR, both at rest and during the narratives, would positively predict PTSS at 1, 3 and 6 months, while HRV indices would be negatively associated. Furthermore, we expected HR measures taken during the trauma narratives to be a stronger predictor of PTSD symptoms than baseline indices.

## Methods

### Participants

Participants derived from a longitudinal study involving 132 children and adolescents (6–13 years) and their primary carers, who were recruited following child exposure to an acute trauma leading to ED attendance (for a description of the overall sample, see Hiller et al. 2018). From this sample, 110 (84%) CYP were offered the opportunity to undergo HR assessments (equipment was not available for the initial 22 families). Of these, 19 (17%) did not want to participate in HR assessments, while for a further seven (6%), the assessment was not performed due to situational restraints (e.g., the family had limited time or the family environment was not conducive to accurate HR data collection). Eight recordings (9.5%) showed too much noise as R-peaks were not clearly identifiable. This led to a final sample of *n* = 76 participants (56.6% of the overall sample) being included in the present analyses. No significant differences were found in age (*p* = 0.48), sex (*p* = 0.38), objective trauma severity (*p* =. 68) and PTSD scores (T1: *p* = 0.70, T2; *p* = 0.39, T3: *p* = 0.15) in the group who completed the HR task vs. those who were not assessed. Compared to the overall eligible sample described in Hiller et al. (2018) (*n* = 341, see supplementary materials for full sample flow chart), there were no significant group differences in child age (*p* = 0.59) or gender (*p* = 0.66). However, those who were included in the final sample had significantly lower triage scores on presenting to the ED (*M* = 1.78 vs. *M* = 2.55, *t*(246) = 5.02, *p* < 0.001) than those not included (see also Hiller et al. 2018). As a lower triage score indicates that medical attention is needed more urgently, children with more severe incidents may be somewhat over-represented in the current sample.

### Measures

#### Descriptive Measures

Descriptive data on family background and child trauma were obtained from parent interviews and ED records. To determine objective trauma severity, triage ratings for urgency of care determined by hospital nurses were used, ranging from *1 = immediate care required* to *4 = less urgent*.

#### University of California PTSD Reaction Index (PTSD-RI), Child Report

Child PTSD symptom-severity at 1, 3 and 6 months was assessed using the child-report of the PTSD- RI (Steinberg et al. 2004), a measure of PTSS for children aged 6 years and older. Children rate the presence of 17 PTSD symptoms over 27-items, according to DSM-IV criteria using a 5-point Likert scale ranging from 0 (*“None of the time*”) to 4 *(“Most of the time”*). The PTSD-RI has been found to differentiate reliably between individuals with and without trauma exposure (Steinberg et al. 2004), and has a high test-rest reliability (Steinberg et al. 2013). Total symptom scores show good internal consistency (in the current study, *Cronbach’s α* = 0.89).

#### Trauma Narratives

Children were asked to provide two narratives of their traumatic experience, always in the same order: the first in the presence of the researcher only (child narrative) and the second together with their parent (joint narrative). For both narratives, instructions asked participants (either the child only, or the parent and child) to begin just before the event occurred and include anything that they thought was important. For the joint narrative, dyads were asked to describe the event together, and the experimenter left the room until called back by the family. Following their initial free recall, participants were provided with some standard, basic prompts to elicit further recollections. These cues contained questions about the child’s thoughts and feelings during and after the event, and were either administered by the experimenter for the child narrative, or by the parents in the joint narrative. There was no time limit on either narrative, and the tasks were audio-recorded. We included both narratives in analyses in order to see whether consistent patterns would be yielded across them.

#### HR Assessments

Electrocardiographic (ECG) data were sampled at a rate of 600 Hz, using the Bio Radio 150 User Unit (Cleveland Medical Devices Inc., Cleveland, OH, USA) and the software “BioCapture” (Biopac-Systems Goleta, CA, USA). Two 2 cm diameter disposable Ag/ AgCl electrodes were placed on the child’s chest, beneath both collar bones, approximately 6–7 in. apart, and a ground electrode was placed on the elbow of the non-dominant hand to allow reliable data collection despite the child potentially moving.

ECG data were pre-processed using the Software “ANSLAB” version 2.6 (Blechert et al. 2016). ECG recordings were visually inspected for artefacts and ectopic beats, and R-peak markers automatically set by the software were manually corrected where necessary. Datasets only displaying high noise, due to equipment failures or electrode detachment, were removed upon visual inspection (see above). Furthermore, as HRV analyses are relatively sensitive to noise distortions (e.g. Bilchick and Berger 2006; Camm et al. 1996), the criterion was set that to allow for reliable analyses, a continuous recording of more than 120 s had to be obtained for the baseline section, and of more than 300 s for each narrative, as the latter were on average substantially longer. This led to the exclusion of 3 subjects at baseline, 1 for the child narrative and 4 for the joint narrative. Furthermore, if more than 3 s of consecutive noise were present, the recording was cut, as reliable reconstruction would not have been possible. Subsequently, the longest “readable” segment was retained. This affected 9 subjects at baseline (with on average 78% of the recording being retained), 10 for the child narrative (77% retained) and 20 for the joint narrative (74% retained for this subset of participants).

After pre-processing, mean HR was extracted, and non-parametric fast Fourier Transformation, using a Welch window-size of 30 s and natural log transformation, was applied to extract indices of HFBP (0.15–4 Hz) and LFBP (0.04–0.15). In a final step, one participant was excluded due to showing an unrealistically high mean HR, and five participants due to exhibiting HFBP and LFBP measures of more than 3 standard deviations above the mean, as these measurements likely reflect recording errors or equipment malfunctions (Minassian et al. 2015). This led to a final sample size of *n* = 70 for baseline analyses, *n* = 74 for the child narrative and *n* = 63 for the joint narrative.

### Procedure

The study was approved by the Research Ethics Committee of the University of Bath and the Oxford A NHS Research Ethics Committee. Eligible families were recruited via four hospital EDs within the UK. Informed consent was obtained from all individual participants included in the study. Assessments were completed at 3 time-points: 1 month post-trauma (T1), and 3 months (T2) and 6 months (T3) later. T1 and T3 assessments took place in the family home, while the shorter T2 assessments were conducted using postal or online questionnaires to reduce assessment burden, unless a face to face assessment was required (e.g., families requested it, younger child age). Parents and children were assessed separately by trained researchers. During the 2 h assessment at T1, parents and children first completed demographic and trauma-related questionnaires. Electrodes for the HR recordings were attached prior to questionnaire completion, and a 3-min baseline recording was obtained while the child completed a simple writing task. Subsequently, HR was recorded for the child narrative task, an anagram task the parent and child completed together (a non-trauma specific, mildly stress-inducing task), and the joint parent-child trauma narrative. During the recordings, the child was instructed to sit as still as possible in order to reduce noise in the recordings. At T2 and T3, parents and children completed a range of questionnaires, including reports on current child PTSD symptoms.

### Data Analytic Strategy

Data were analysed using SPSS Version 22. A square root transformation was applied to UCLA-RI scores to address skewness. Correlational analyses were performed to determine basic associations between variables. Subsequently, linear regression analyses were used to investigate whether HR indices (mean HR, LFBP and HFBP) predicted child PTSD symptoms at T1, T2 and T3. Residualized change scores from baseline HR to task HR were calculated for both narratives to control for pre-existing baseline HR differences. Shapiro-Wilk tests indicated these change scores were normally distributed. Since HR indices may be associated with each other, each HR predictor was entered into a separate regression model. Where T1 HR indices were found to predict symptoms at T2 or T3, analyses were re-run controlling for T1 symptom severity, to determine whether HR has predictive power above and beyond initial symptoms. Age, sex and triage were considered as potential control variables for all analyses. The Benjamini-Hochberg procedure (Benjamini and Hochberg 1995) with a false discovery rate set to 5% was used to control to control for multiple testing. All findings remained significant.

## Results

### Descriptive Statistics

The sample comprised 76 children (46 male) aged 6 to 13 years. The majority of children had experienced a road traffic accident or other accidental injury as their index trauma. Accompanying parents were predominantly mothers, aged 26–60 years. Detailed child and parental descriptive data are provided in Table 1.

**Table 1:** Descriptive information

Demographic characteristics	Statistic (*N* = 76)
Parent characteristics	
Age in years, *M*(*SD*)	39.82 (6.53)
Proportion mothers, *N(%)*	70 (92.1%)
Proportion married or cohabiting *N(%)*	51 (67.1%)
Education Status, N(%):	
School until 18 years or younger	35 (47.3%)
Further Education (Vocational Training/Diplomas,…)	16 (21.6%)
Higher Education	23 (31.1%)
Child characteristics	
Age in years *M, (SD)*	10.05 (0.22)
Proportion male, *N(%)*	46 (60.5%)
Ethnicity- Caucasian, *N(%)*	68 (89.5%)
Triage Category, N(%):	
1 (immediate attention required)	37 (48.7%)
2 (very urgent)	16 (21.1%)
3 (urgent)	13 (17.1%)
4 (less urgent)	10 (13.2%)
Days in Hospital (Min-Max, *M (SD*))	0–28, 2.80 (5.40)
Days of school missed (Min-Max, *M (SD)*)	0–28, 5.15 (6.22)
Proportion requiring ambulance/ helicopter, *N(%):*	54 (71.0%)

Child age, sex and objective trauma severity were explored as potential covariates. Results showed that age was negatively associated with PTSS at T1 and T3 (PTSD-RI T1: *r* = −0.33, *p* < 0.001, PTSD-RI T3: *r* = −0.27, *p* < 0.05), as well as with the following HR indices: baseline HR (*r* = −0.28, *p* < 0.05), joint narrative HR (*r* = −0.27, *p* < 0.05) and joint narrative LFBP (*r* = 0.23, *p* < 0.05). No significant associations with either PTSD or HR measures were found for sex or triage category. Only child age was therefore controlled for in regression analyses.

### Associations Between HR Indices and PTSD

Mean child scores on the main variables of analysis are provided in Table 2, while correlations between the main variables are presented in Table 3. Preliminary correlational analyses revealed that none of the HR measures obtained at baseline were associated with PTSS at any time point. Consequently, baseline HR as a predictor of PTSS was not explored further in regression analyses. For the child and joint narratives, both cross-sectional and longitudinal correlations between mean HR/ HRV residualized change scores and PTSS were identified (see Table 3), which were further tested in linear regression analyses, controlling for child age.

**Table 2:** Child mean scores on main outcome measures

Child variable	Mean	*SD*	95% *CI*: lower bound; upper bound
PTSD-RI- T1	18.76	12.54	15.90; 21.63
PTSD-RI- T2	15.44	12.82	12.19; 18.69
PTSD-RI- T3	13.91	12.59	10.86; 16.95
Baseline Mean HR	90.07	10.17	87.76; 92.37
Baseline HFBP	7.67	1.08	7.42; 7.91
Baseline LFBP	8.91	0.84	8.69; 9.11
Child Narrative Mean HR	92.00	10.57	89.52; 94.43
Child Narrative LFBP	7.85	0.86	7.66; 8.04
Child Narrative HFBP	9.32	0.78	9.16; 9.49
Joint Narrative Mean HR	89.25	9.00	87.01; 91.46
Joint Narrative LFBP	8.03	0.81	7.83; 8.22
Joint Narrative HFBP	9.61	0.64	9.45; 9.76

Note: PTSD-RI T2 scores are based on *N* = 59 and T3 scores on *N* = 68 due to participant dropout, all other scores are based on *N* = 76 at T1

*SD* standard deviation, *HR* heart-rate, *HFBP* high frequency band power, *LFBP* low frequency band power, *CI* confidence interval, *PTSD-RI* posttraumatic stress disorder reaction index

**Table 3:** Associations between PTSD symptoms at T1, T2 and T3 and heart-rate indices at 1 month post-trauma

	1.	2.	3.	4.	5.	6.	7.	8.	9.	10.	11.	12.
PTSD												
1. T1 symptoms	1.00											
2. T2 symptoms	0.51^**^	1.00										
3. T3 symptoms	0.50^**^	0.67^**^	1.00									
Mean HR												
4. Baseline	0.10	0.22	0.20	1.00								
5. Child narrative	0.33^**^	0.36^**^	0.24	0.06	1.00							
6. Joint narrative	0.27^*^	0.44^**^	0.22	0.07	0.63^**^	1.00						
HFBP												
7. Baseline	0.03	−0.19	−0.14	−0.70^**^	0.20	0.14	1.00					
8. Child Narrative	−0.29^*^	−0.32^*^	−0.32^*^	−0.23	−0.59^**^	−0.21	0.06	1.00				
9. Joint Narrative	−0.35^**^	−0.38^**^	−0.33^*^	−0.12	−0.39^**^	−0.41^**^	0.03	0.66^**^	1.00			
LFBP												
10. Baseline	0.09	−0.02	−0.10	−0.49^**^	0.12	0.14	0.74^**^	0.07	−0.08	1.00		
11. Child Narrative	−0.25^*^	−0.37^*^	−0.25	−0.32^**^	−0.33^**^	−0.24	0.16	0.64^**^	0.55^**^	−0.02	1.00	
12. Joint Narrative	−0.40^**^	−0.35^*^	−0.36^**^	−0.40^**^	−0.35^**^	−0.37^**^	0.23	0.48^**^	0.70^**^	−0.04	0.64^**^	1.00

*HR* heart-rate, *HFBP* high frequency band power, *LFBP* low frequency band power. Heart-rate scores for the narratives are based on residualized change scores between baseline and the respective narrative

* *p* < 0.05, ** *p* < 0.01

### Cross-Sectional Analyses at T1

For both narrative tasks, correlational analyses revealed change in mean HR to be positively associated with T1 PTSS; similarly, changes in LFBP/HFBP were inversely associated with T1 PTSS (Table 3). We explored these associations further, using linear regression analyses to examine whether HR and HRV measures were cross-sectionally associated with child PTSS at T1, controlling for child age. For the child narrative (Table 4), regression analyses found that, in accordance with our hypotheses, residualized change scores for mean HR were positively associated with T1 PTSS (*R*^*2*^ change compared to a model including age only = 0.09, *p* = 0.008), whereas HFBP power was inversely associated with T1 PTSS (*R*^*2*^ change = 0.09, *p* = 0.011). However, the effect of LFBP was not significant once age was controlled for (*R*^*2*^ change = 0.04, *p* = 0.102). For the joint narrative, mean HR positively (*R*^*2*^ change = 0.06, *p* = 0.045) and HFBP (*R*^*2*^ change = 0.18, *p* < 0.001) and LFBP (*R*^*2*^ change = 0.12, *p* = 0.004) negatively predicted symptoms, with small to medium effect sizes (see Table 5).

**Table 4:** Regression analyses: prediction of T1, T2 and T3 PTSD symptoms from age and heart-rate change indices for child narrative

Model, predictors	T1 (1 month post-trauma)	T2 (3 month follow-up)	T3 (6 months follow-up)
Model 1	*R*^*2*^ = 0.13, F(2,66) = 6.28**	*R*^*2*^ = 0.10, F(2,52) = 4.07*	*R*^*2*^ = 0.08, F(2,59) = 3.50*
Age	*B* = −0.22 (0.10), *β* = −0.26*	*B* = −0.11 (0.11), *β* = −0.13	*B* = −0.26 (0.13), *β* = −0.25*
Mean HR	*B* = 0.47 (0.21), *β* = 0.26*	*B* = 0.58 (0.24), *β* = 0.32*	*B* = 0.36 (0.13), *β* = 0.17
Model 2	*R*^*2*^ = 0.15, F(2,65) = 7.20**	*R*^*2*^ = 0.10, F(2,52) = 3.95*	*R*^*2*^ = 0.12, F(2,59) = 5.01**
Age	*B* = −0.24 (0.10), *β* = −0.29*	*B* = −0.15 (0.11), *β* = −0.17	*B* = −0.27 (0.12), *β* = −0.27*
HFBP	*B* = −0.51 (0.20), *β* = −0.29*	*B* = −0.24 (0.10), *β* = −0.31*	*B* = −0.56 (0.26), *β* = −0.26*
Model 3	*R*^*2*^ = 0.10, F(2,66) = 4.90**	*R*^*2*^ = 0.09, F(2,52) = 3.81*	*R*^*2*^ = 0.09, F(2,59) = 4.14*
Age	*B* = −0.23 (0.10), *β* = −0.28*	*B* = −0.11 (0.11), *β* = −0.13*	*B* = −0.25 (0.13), *β* = −0.24
LFBP	*B* = −0.32 (0.19), *β* = −0.19	*B* = −0.53 (0.23), *β* = −0.31*	*B* = −0.44 (0.25), *β* = −0.22

*HR* heart-rate, *HFBP* high frequency band power, *LFBP* low frequency band power. For all HR indices, change scores from baseline to narrative were used

* *p* < 0.05, ** *p* < 0.01

**Table 5:** Regression analyses: prediction of T1, T2 and T3 PTSD symptoms from age and heart-rate change indices for joint narrative

Model, predictors	T1 (1 month)	T2 (3 months)	T3 (6 months)
Model 1	*R*^*2*^ = 0.13, F(2,56) = 5.30**	*R*^*2*^ *= 0.19, F(2,45) = 14.65*	*R*^*2*^ = 0.09, F(2,51) = 3.65*
Age	*B* = −0.20 (0.11), *β* = −0.24	*B* = −0.06 (0.11), *β* = −0.07	*B* = −0.22 (0.13), *β* = −0.24
Mean HR	*B* = 0.46 (0.22), *β* = 0.26*	*B* = 0.70 (0.22), *β* = 0.44 **	*B* = 0.38 (0.26), *β =* 0.20
Model 2	*R*^*2*^ = 0.19, F(2, 56) = 7.82**	*R*^*2*^ = 0.20, F(2, 45) = 6.84**	*R*^*2*^ = 0.18, F(2,51) = 6.76**
Age	*B* = −0.26 (0.10), *β* = −0.31*	*B* = −0.18 (0.11), *β* = −0.23	*B* = −0.27 (0.12), *β* = −0.29*
HFBP	*B* = −0.62 (0.21), *β* = −0.35**	*B* = −0.75 (0.22), *β* = −0.44**	*B* = −0.67 (0.24), *β* = −0.35**
Model 3	*R*^*2*^ = 0.26, F(2, 56) = 11.29**	*R*^*2*^ = 0.14, F(2, 45) = 4.76*	*R*^*2*^ = 0.21, F(2,51) = 7.97**
Age	*B* = −0.18 (0.10), *β* = −0.21	*B* = −0.10 (0.11), *β* = −0.12	*B* = −0.19 (0.12), *β* = −0.20
LFBP	*B* = −0.82 (0.21), *β* = −0.45**	*B* = −0.65 (0.24), *β* = −0.37*	*B* = −0.80 (0.25), *β* = −0.40**

*HR* heart-rate, *HFBP* high frequency band power, *LFBP* low frequency band power. For all HR indices, change scores from baseline to narrative were used

* *p* < 0.05, ** *p* < 0.01

### Predicting PTSD Symptoms at T2

Basic correlational analyses for T2 PTSS (i.e., 3 month follow-up) revealed that T1 mean HR was positively associated with child/ adolescent PTSS, whereas LFBP/HFBP showed inverse associations, for both the child and joint narratives (Table 3). Again, regression was used to probe these effects further. For the child narrative, when performing regression analyses controlling for age, residualized change scores for mean HR positively predicted T2 PTSS (*R*^*2*^ change = 0.11, *p* = 0.014), while HFBP (*R*^*2*^ change = 0.10, *p* = 0.021) and LFBP (*R*^*2*^ change = 0.09, *p* = 0.023) were negatively associated (Table 4). Age was only a significant predictor of PTSS in the model including LFBP, where it showed a negative association. Similarly, for the joint narrative, residualized change scores for mean HR positively predicted PTSS (*R*^*2*^ change = 0.18, *p* = 0.003), and for HFBP (*R*^*2*^ change = 0.19, *p* = 0.002) and LFBP (*R*^*2*^ change = 0.11, *p* = 0.018) negatively predicted PTSS when controlling for age, which was a non-significant predictor in all models (see Table 5).

We next re-ran the models of T2 PTSS, this time including T1 PTSS as an additional predictor, in order to see whether T1 HR indices had any longitudinal predictive effect above and beyond initial distress. Following the addition of T1 PTSS scores to the regression model (*B* = 0.46, *SE* = 0.12, *β* = 0.47, *p* = 0.001), mean HR assessed during the T1 joint narrative continued to be a positive predictor of T2 PTSS (*B* = 0.46, *SE* = 0.20, *β* = 0.29, *p* = 0.003; *R*^*2*^ change = 0.07, *p* = 0.030), model *R*^*2*^ = 0.37, *F* (3,44) = 10.12, *p* < 0.001. However, all other predictive effects of child narrative and joint narrative HR indices were rendered non-significant by the inclusion of T1 PTSS in the model (*βs* ranged −0.22 to 0.20, all *p* > 0.07).

### Predicting PTSD Symptoms at T3

Hierarchical regression models were only run for those T1 HR indices showing significant correlations with child PTSS at T3 (i.e., 6 month follow-up) (see Table 3). For the child narrative, residualized change scores for HFBP significantly negatively predicted T3 symptoms when entered in a regression model controlling for age (*R*^*2*^ change = 0.07, *p* = 0.037; see Table 4). Similarly, for the joint narrative, residualized change scores were significantly negatively associated with child PTSS for HFBP (*R*^*2*^ change = 0.15, *p* = 0.003) and LFBP (*R*^*2*^ change = 0.10, *p* = 0.017) (see Table 5 for full statistics). When including T1 PTSS scores in regression models, all effects were rendered non-significant (*β*s ranged −0.13 to −0.09, all *p* > 0.25). Thus, for all models at step 2, T1 PTSS was the only significant predictor of T3 outcomes (25.5% of variance explained by itself).

## Discussion

We examined whether mean HR and HRV measures taken 1 month after exposure to a traumatic event are reliable predictors of child PTSS, both concurrently and at 3 and 6 months follow-ups. We found that baseline HR at 1 month was not correlated with PTSS cross-sectionally or longitudinally. By contrast, indices of HR reactivity in response to trauma provocation (either child only or joint narrative) showed a relatively reliable pattern of correlations with symptoms cross-sectionally, and longitudinally at 3 month follow-up, with less robust prediction of 6 month outcomes.

We found no evidence that baseline HR parameters measured at 1 month post-trauma are associated with concurrent levels of PTSS in CYP. This is consistent with previously reported null findings in relation to basal HR and child PTSS in children (Jones-Alexander et al. 2005; Kirsch et al. 2015), but is in contrast to the adult literature where basal elevations in sympathetic activity have commonly been reported (Pole 2007). It is conceivable that mental health linked lifestyle factors (e.g., increase tobacco and alcohol use) partially explain PTSD related changes in basal HR in the adult literature. Moreover, the adult field has disproportionately reported on veteran samples, where chronic stress and trauma exposure are more typical (Pole 2007), whereas we focused on children exposed to acute, single incident trauma. It will be important to conduct more long term studies of resting HR in relation to child PTSD, in order to examine whether changes emerge over time and could contribute to evidence of increased risk of cardiovascular disease in association with adverse childhood experiences (Danese and McEwen 2012; Suglia et al. 2017).

We also found no evidence that baseline HR as measured at 1 month post-trauma was predictive of PTSS 3 and 6 months later. This contrasts with evidence that mean HR shortly following ED admission positively predicts long-term PTSS (Kassam-Adams et al. 2005; Alisic et al. 2011). The current null findings should be treated cautiously, as our study was not well powered to detect longitudinal effects of the magnitude previously reported in the literature (*r* = 0.18, a small effect, based on meta-analysis by Alisic et al. 2011). However, the greater elapsed time since trauma exposure is one likely explanation for the lack of prediction in the current study. Theoretical accounts of longitudinal associations between elevated post-trauma HR and later PTSS highlight the potential for adrenergic activation to augment memory consolidation/conditioning for trauma stimuli as the putative underlying mechanism (Bryant 2006), but the same account cannot easily be applied to HR parameters 1 month following trauma. Future studies that complete HR assessments longitudinally, starting from ED presentation, can help to address such questions relating to underlying mechanisms, and can identify optimal time-points at which risk marker assessments for PTSS should be conducted (Olsson et al. 2008).

In contrast to null findings for baseline HR, when we examined reactivity to trauma cues using two narrative tasks, relatively higher mean HR as compared to baseline was cross-sectionally associated with child/adolescent PTSS at 1 month post trauma with small to medium effect sizes, for both narratives. Effects were somewhat stronger for the “parent-child” than the “child only” narrative, and it is possible that the joint narrative resulted in higher emotional involvement of the child, and consequently a pattern of HR reactivity that more strongly associated with PTSS. However, the child narrative was always conducted first, meaning that order effects may equally account for the pattern of findings. Overall, our observations challenge previous studies which found no differences in mean HR between CYP with PTSD and trauma controls when listening to idiosyncratic trauma scripts (Jones-Alexander et al. 2005; Kirsch et al. 2015). The current findings are consistent with the observations of one previous study of pre-school children, which found higher autonomic responding (stronger reduction in RSA) to trauma cues in those with PTSD (Gray et al. 2018), and indicate that physiological reactivity to trauma cues in association with PTSD in CYP may show a profile more similar to that seen in adults than previously thought.

We also found evidence that indices of HRV are cross-sectionally associated with PTSS in young people with small to medium effect sizes, specifically HFBP as an indicator of the PNS response (De Bellis and Putnam 1994), and LFBP as a putative indicator of SNS-PNS balance (Nagpal et al. 2013). Relative reductions in HRV change scores from baseline during the narratives at 1 month post-trauma were negatively associated with PTSS. Lower HRV has been linked to a diminished self-regulation (Kim et al. 2018; Kemp and Quintana 2013), which makes our findings consistent with an overall dysregulated ANS response to trauma cues. The current study thus lays a promising basis for further explorations of the role of HRV as a child/ adolescent PTSD risk-marker. Importantly, while the results in the current study were upheld when correcting for multiple testing using the Benjamini Hochberg procedure, replication using larger samples and applying more stringent criteria to control for multiple testing is needed.

Analyses of longitudinal associations between physiological reactivity to each of our trauma narratives at T1 and PTSS at 3- and 6-month follow-up, showed patterns of associations that largely replicated cross-sectional findings, although effects were somewhat less robust for the longer follow-up. Despite these observations suggesting that altered physiological reactivity could be a marker for persistent distress, only HR reactivity during the joint narrative at T2 predicted later PTSS (medium effect size) once T1 symptom levels were controlled for. This is not entirely surprising, as PTSS at 1-month post-trauma are one of the strongest predictors of long-term PTSD risk in CYP (Trickey et al. 2012). Furthermore, according to our cross-sectional analyses, HR indices and PTSD symptom measures obtained at 1 month have a substantial amount of common variance, potentially leading to a decreased predictive validity of HR indices when these two potential risk-markers are combined. Thus, co-varying for concurrent symptoms in trying to predict later distress is a particularly stringent test of causality. Nonetheless, based on the current findings it is possible that physiological reactivity to trauma cues is a marker for concurrent distress, rather than a causal factor in the aetiology of PTSD.

Our study has several strengths, including a longitudinal design, the incorporation of both baseline and trauma reactivity measures, and sensitive measurement of HR and HRV. Nonetheless, findings must be considered in the light of several limitations. First, as the HR measurements were only introduced several months into the study, and a number of recordings had to be excluded due to high noise, the sample size was only moderately large, potentially limiting our power to detect long-term effects. Second, while the age range was smaller than in many previous studies, the sample still comprised mixed developmental stages. With mean resting HRs changing substantially over the course of development (Kassam-Adams et al. 2005), further studies are required to investigate age effects in more detail and derive reference values before HR assessments can be established as routine clinical markers. Third, the current study did not take into account child pre- and post-traumatic medical/physical health status, which could have potentially confounded heart-rate measures. Fourth, CYP in the current sample had all experienced single-incident traumas. This limits the generalizability of current findings to other populations, such as children exposed to repeated or prolonged traumas, or those living in high-risk contexts, who might exhibit different patterns of HR reactivity following trauma exposure (Cloitre et al. 2009). Relatedly, symptoms were generally low at T3, with few children showing clinically significant PTSS levels, which may have contributed to the lack of strong predictive effects. It may be of benefit to replicate the study in a clinical sample, investigating the association between HR indices and PTSD diagnoses, which was not possible here due to small numbers meeting diagnostic criteria. Finally, it is important to note that there was a slight over-representation of children with more severe injuries in our sample, as compared to the overall eligible sample, with findings requiring confirmation in a sample that does not show this bias.

In sum, the current study extended present research on HR indices as a risk-marker for child and adolescent PTSD in several directions, including an investigation of the predictive value of HRV assessments, which had not previously been studied in this population. Extending our knowledge on this matter may ultimately lead to improved routine assessments, as well as the targeted implementation of preventive and therapeutic measures, with the aim of buffering adverse outcomes following child and adolescent trauma exposure as effectively as possible.

## Electronic supplementary material

ESM 1 (DOCX 45 kb)
