# Supplementary material for: A Longitudinal Examination of Heart-Rate and Heart Rate Variability as Risk Markers for Child Posttraumatic Stress Symptoms in an Acute Injury Sample
Source: J Abnorm Child Psychol. 2019 May 10;47(11):1811–20. doi: 10.1007/s10802-019-00553-2 (PMC6805807; doi:10.1007/s10802-019-00553-2)
Supplement: Supplementary file 1 — (DOCX 45 kb) [file 10802_2019_553_MOESM1_ESM.docx]

S1. *Flow chart of recruitment numbers (see Hiller et al., 2018)*

341 eligible families

Reasons for non-contact:

- 94 could not be contacted within 1-month

- 52 were not interested/too busy

- 2 believed it would be too distressing for

child

194 contacted by research team

Reasons for not participating

- 25 could not have their first assessment scheduled within 1-month

- 13 were not interested/too busy

- 5 believed it would be too distressing for

child

151 agreed to participate

19 families could not be assessed within required 1-month post-hospital timeframe.

Full Study Sample at T1: *N* = 132

Heart-rate data missing:

- 22 equipment not available

- 19 did not want to take part

- 6 due to situational constraints

- 8 traces removed due to noise

Heart-Rate Sample at T1: *N* = 76

8 families out of 76 could not be contacted or declined

17 out of 76 questionnaires not returned within timeframe

6-month follow-up: *n* = 68

(89.5% of original sample)

3-month follow-up: *n* = 59

(77.6 % of original sample)
